# *Primulina acutiloba* (Gesneriaceae), a new species from Northwest Guangxi, China

**DOI:** 10.3897/phytokeys.272.178656

**Published:** 2026-03-18

**Authors:** Chi Xiong, Jia-Xin Fu, Qi Qin, Bo Pan, Hong Liu, Fang Wen

**Affiliations:** 1 Hubei Provincial Key Laboratory for Protection and Application of Special Plant Germplasm in Wuling Area of China, Key Laboratory of State Ethnic Affairs Commission for Biological Technology, College of Life Sciences, South-Central Minzu University, Wuhan 430074, China Gesneriad committee of China Wild Plant Conservation Association, Guangxi Institute of Botany, Guangxi Zhuang autonomous Region and Chinese Academy of Science Guilin China; 2 Guangxi Key Laboratory of Plant Conservation and Restoration Ecology in Karst Terrain, Guangxi Institute of Botany, Guangxi Zhuang Autonomous Region and Chinese Academy of Sciences, Guilin 541006, China Guangxi Key Laboratory of Plant Conservation and Restoration Ecology in Karst Terrain, Guangxi Institute of Botany, Guangxi Zhuang Autonomous Region and Chinese Academy of Sciences Guilin China; 3 Gesneriad Conservation Center of China (GCCC), National Gesneriaceae Germplasm Resources Bank of GXIB, Gesneriad committee of China Wild Plant Conservation Association, Guangxi Institute of Botany, Guangxi Zhuang autonomous Region and Chinese Academy of Science, Guilin 541006, China South-Central Minzu University Wuhan China; 4 Luojiang Middle School, Deyang 618500, China Luojiang Middle School Deyang China

**Keywords:** Baise, Guangxi, limestone, new taxon, taxonomy

## Abstract

A new species of Gesneriaceae, *Primulina acutiloba* C.Xiong, J.X.Fu & F.Wen, from the limestone karsts of Northwest Guangxi, China, is described and illustrated. Morphologically, it resembles *Pri. pingguoensis* H.S.Ma & B.Pan and *Pri. pseudoeburnea* (D.Fang & W.T.Wang) Mich. Möller & A. Weber in corolla shape and coloration, particularly in the presence of purple stripes extending from the throat to the lobes. However, it can be readily distinguished by a combination of characters, including the shape of the leaf blades, bracts, and calyx lobes, the length of the peduncle and pistil, and the height of the disc. Currently, only a single population is known from the type locality, comprising approximately 60–80 mature individuals. Following the IUCN Red List Categories and Criteria, the species is provisionally assessed as Endangered (EN C2 b).

## Introduction

*Primulina* Hance is one of the most species-rich genera within Gesneriaceae, comprising more than 250 species (GRC 2026). The genus is primarily distributed in limestone regions and Danxia landforms of southern, southwestern, and eastern China, extending to north-central Vietnam (Weber et al. 2011a; Xu et al. 2021). China represents the center of diversity for *Primulina*, harbouring at least 237 species (GRC 2026). The genus exhibits a high degree of endemism and extremely narrow distribution ranges, often characterized as ‘one cave, one species’ or ‘one hill, one species’ (Monro et al. 2018; Fu et al. 2022; Wei 2023). Over the past five years, the rate of taxonomic discovery has increased, with approximately eight new species being described annually (GRC 2026; Wen et al. 2026).

In December 2024, during a survey of vascular plants in karst caves in Baise city, northwestern Guangxi, we discovered an unknown species at a single location. At the time of collection, the plants were sterile, lacking flowers or fruits, but their succulent, rosulate leaves suggested an affinity with Gesneriaceae, most likely within *Primulina*. Several living plants were collected and cultivated at the Gesneriad Conservation Center of China (GCCC) and the National Gesneriaceae Germplasm Bank of GXIB (NGGRB) for further study.

The cultivated plants flowered in August 2025, producing pale purple flowers with acute corolla lobes and purple stripes extending from the throat to the lobes. These floral features are relatively uncommon in *Primulina* but resemble those found in certain species of *Petrocodon* Hance, such as *Pet. asterostriatus* F.Wen, Y.G.Wei & W.C.Chou (Yang et al. 2022), *Pet. ionophyllus* F.Wen, S.Li & B.Pan (Li et al. 2020), and *Pet. integrifolius* (D.Fang & L.Zeng) A.Weber and Mich. Möller (Weber et al. 2011b). However, detailed examination of vegetative and reproductive morphology supports its placement within *Primulina*. Diagnostic features include succulent, opposite leaves often appearing as a whorl of four (versus sub-coriaceous, alternate leaves in *Petrocodon*), a relatively short cylindric to infundibular corolla tube (rather than the narrowly cylindric tube typical of *Petrocodon*), two stamens with cohering anthers, and a chiritoid stigma, a feature rarely observed in *Petrocodon*.

The corolla shape and coloration, particularly the purple stripes extending from the throat to the lobes, resemble those of *Pri. pingguoensis* H.S.Ma & B.Pan (Ma et al. 2024), and *Pri. pseudoeburnea* (D.Fang & W.T.Wang) Mich. Möller and A.Weber (Weber et al. 2011a, 2011b), both of which also occur in Baise City. Nevertheless, detailed comparison of morphological characters, including the leaf blades, calyx lobes, corolla, disc, and pistil, clearly distinguishes the plants from these two congeners. Based on this comprehensive morphological evidence, the specimens are here described as a new species of *Primulina*.

## Materials and methods

Approximately ten living plants were collected during fieldwork in Tianyang district, Baise city, Guangxi, China, in December 2024. These individuals were subsequently cultivated in the nurseries of GCCC and NGGRB in Guilin city. Voucher specimens prepared from the cultivated material were stored at IBK (Guangxi Institute of Botany), following herbarium acronyms as standardized by the Index Herbariorum (Thiers 2025).

The species description was based on observation of cultivated material, including six flowering individuals. Photographs were taken using a Nikon D7200 digital camera (Japan, Nikon Co., Ltd.). Morphological characters were examined from both living and herbarium specimens using an Olympus SZX16 binocular microscope (Japan, Olympus Co., Ltd.). Terminology follows Beentje (2016).

## Taxonomic treatment

### Primulina acutiloba

Taxon classificationPlantaeLamialesGesneriaceae

C.Xiong, J.X.Fu & F.Wen sp. nov.

F6A57D8F-428D-5B69-AB47-E8990DD5F9C9

urn:lsid:ipni.org:names:77377812-1

Figs 1, 2

#### Type.

China • Guangxi Zhuangzu Autonomous Region: cultivated in Guilin Botanical Garden, introduced from Baise city, Tianyang district, Dongjing town, Wanlao village, 23°41'N, 106°38'E, elev. ca. 480 m, 18 August 2025, *Chi Xiong et al. XC240818-01* (**holotype**IBK!, IBK00471733; **isotypes**IBK!, IBK00471734, 00471735).

**Figure 1.**
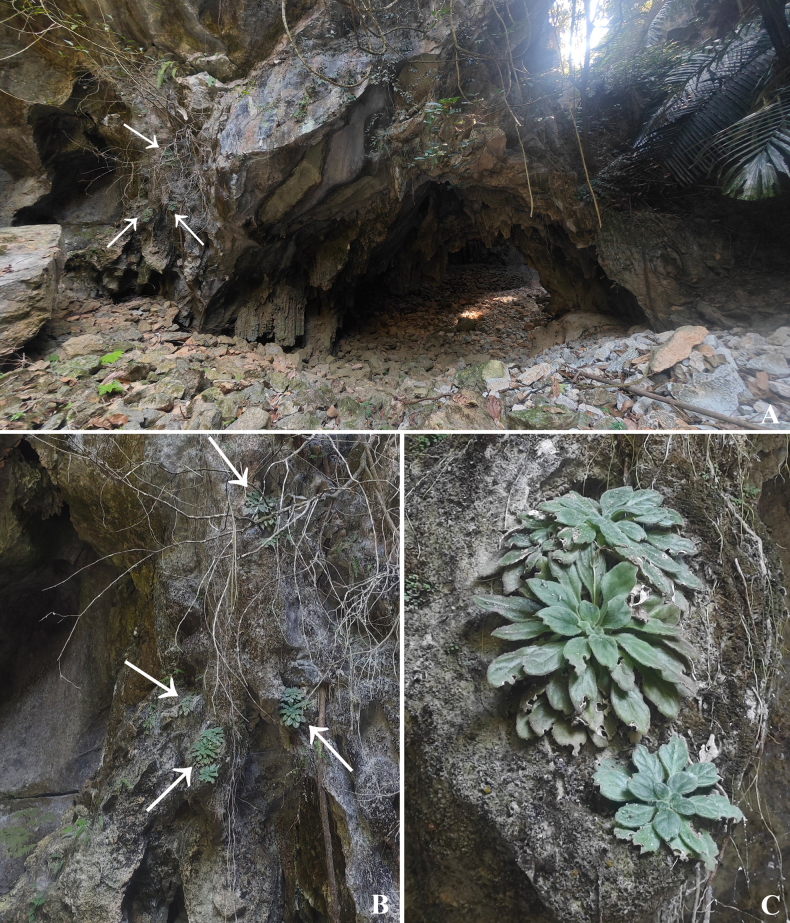
Habitat of *Primulina acutiloba* C.Xiong, J.X.Fu & F.Wen, sp. nov. **A, B**. Habitat, arrows indicate plants; **C**. Plant habit. (All photographed by Chi Xiong).

**Figure 2.**
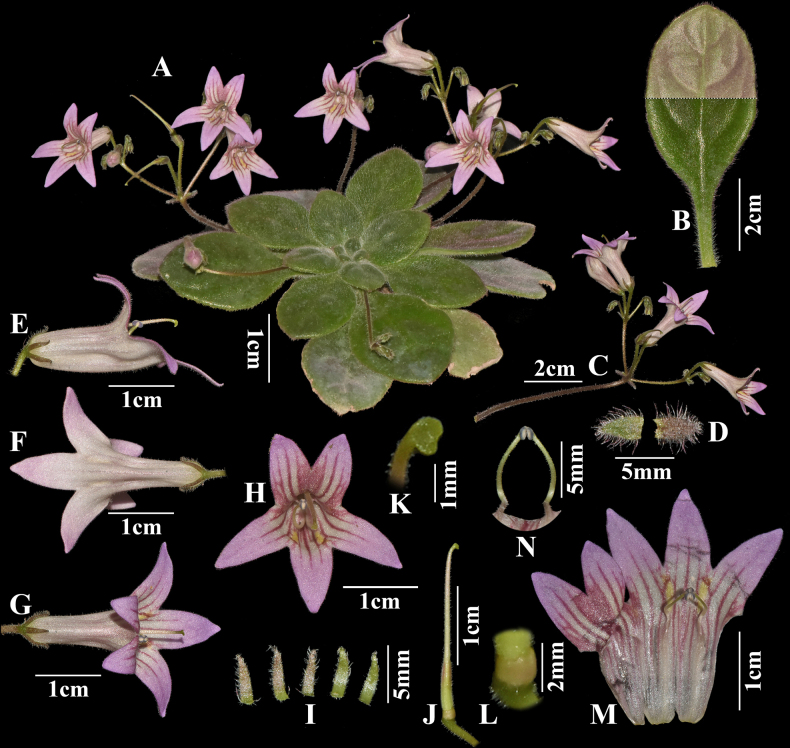
*Primulina acutiloba* C.Xiong, J.X.Fu & F.Wen, sp. nov. **A**. Flowering plant cultivated in GCCC; **B**. Abaxial (above) and adaxial (below) sides of leaf blade; **C**. Cymes (arrow shows bracts); **D**. Bracts, dissected; **E**. Side view of flower; **F**. Ventral view of flower; **G**. Dorsal view of flower; **H**. Front view of flower; **I**. Sepals, dissected; **J**. Pistil and disc; **K**. Stigma; **L**. Disc; **M**. Opened corolla showing stamens and staminodes; **N**. Stamens. (All photographed by Chi Xiong).

#### Diagnosis.

**Figure 3.**
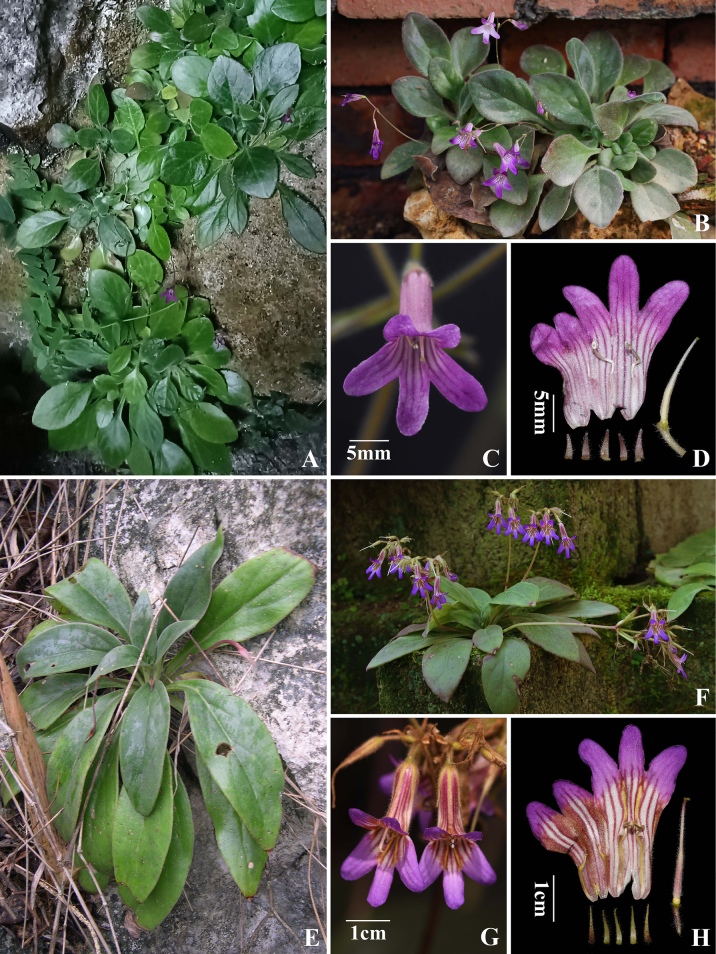
*Primulina pingguoensis* (**A–D**) and *Pri. pseudoeburnea* (**E–H**). **A, E**. Plant in natural habitat; **B, F**. Flowering plant cultivated in GCCC; **C, G**. Flowers; **D, H**. Floral anatomy. (**A, C, E**. Photographed by Fang Wen; **B, F**. By Chi Xiong; **D, G, H**. By De-Chang Meng).

Within *Primulina*, *Pri. acutiloba* is readily distinguished by its triangular corolla lobes with prominent stripes. In overall corolla shape, coloration, and the presence of purple stripes extending from the throat to the lobes, it resembles *Pri. pingguoensis* and *Pri. pseudoeburnea* (Fig. 3). However, it differs from both species in having peduncle 3–8 cm long (vs. 8–16.5 cm in *Pri. pingguoensis* and 10–19 cm in *Pri. pseudoeburnea*), elliptic bracts (vs. linear or linear-lanceolate in *Pri. pingguoensis* and lanceolate in *Pri. pseudoeburnea*), corolla 25–28 mm long (vs. ca. 16–20 mm in *Pri. pingguoensis* and ca. 30 mm in *Pri. pseudoeburnea*), and pistil 21–24 mm long (vs. ca. 11–15 mm in *Pri. pingguoensis* and ca. 20 mm in *Pri. pseudoeburnea*).

#### Description.

Perennial herbs. ***Rhizome*** subterete, 2–10 cm long, ca. 1 cm in diameter, simple, becoming longer and thicker with age. ***Leaves*** 12–35, opposite, all basal, clustered at the rhizome apex, petiolate; petiole 1–3 cm long, ca. 2 mm in diameter, light green, densely pubescent; leaf blade narrowly elliptic, elliptic to ovate, 2.2–6.5 × 1.2–3.2 cm, succulent to thickly chartaceous, adaxially dark green to green, abaxially light green, densely white villous on both surfaces, sometimes purple villous in upper half; base cuneate to broadly cuneate; margin entire or sometimes obscurely and shallowly serrate; apex acute to obtuse; lateral veins 3–6 on each side. ***Cymes*** axillary, 2–4, 3–12 flowers per cyme; peduncle 3–8 cm long, ca. 1.5 mm in diameter, pubescent; ***bracts*** 2, opposite, elliptic, 3.5–4.2 × 1.5–1.8 mm, villous abaxially, glabrous adaxially; margin entire; apex obtuse. ***Calyx*** 5-parted to base, lobes lanceolate, 4.0–4.5 × 0.9–1.2 mm, outside light purple, pubescent, inside yellow-green, nearly glabrous; margin entire; apex acute. ***Corolla*** pale purple, 2.5–2.8 cm long, with two yellow patches and ca. 15 purple stripes on adaxial surface of throat and lobes, outside glandular and glandular puberulent, inside glabrous, tube nearly cylindric to infundibular, 1.2–1.5 cm long, 7–8 mm in diameter at throat, laterally compressed at bottom half, ventrally slightly carinate; limb distinctly 2-lipped; adaxial lip 2-parted to middle, lobes triangular, apex acute, 6.5–7.5 × 5.0–5.5 mm, abaxial lip 3-parted to near base, lobes narrowly triangular, apex acute, 8.5–11.5 × 5.2–6.0 mm. ***Stamens*** 2, adnate ca. 11 mm above corolla base; ***filaments*** ca. 6 mm long, pale greenish-yellow, slightly sigmoid, glandular puberulent; ***anthers*** dark purple externally, reniform, slightly constricted at middle, with the dehiscence slit white, ca. 1 mm long; ***staminodes*** 3, glabrous, white, lateral ones linear, 1.5–1.8 mm long, apex capitate, glabrous, adnate to ca. 8 mm above corolla tube base; central one inconspicuous, adnate near corolla tube base. ***Disc*** annular, ca. 1.2 mm high, margin repand, greenish-yellow, glabrous. ***Pistil*** 2.1–2.4 cm long; ***ovary*** 7–8 mm long, ca. 1.5 mm in diameter, yellowish green to brownish green, glandular-pubescent; ***style*** ca. 1.5 cm long, 0.5 mm in diameter, pale green, glandular-pubescent; ***stigma*** chiritoid, obtrapeziform, ca. 1 mm long, green, apex shallowly 2-lobed. Fruit and seeds not seen.

#### Phenology.

Flowering from August to October.

#### Etymology.

The specific epithet ‘*acutiloba*’ is derived from the Latin *acutus* (meaning ‘acute’) and *lobus* (meaning ‘lobe’), referring to the characteristically acute apices of the corolla lobes.

#### Vernacular name.

jiān bàn bào chūn jǔ tái (Chinese pronunciation); 尖瓣报春苣苔 (Chinese name).

#### Distribution and ecology.

The new species is currently found only from the type locality at an elevation of approximately 480 m. It grows on calcified cliffs beneath low shrubs (Fig. 1A, B), in association with species such as *Arenga westerhoutii* Griff. (Arecaceae) and *Ficus tinctoria* subsp. *gibbosa* (Blume) Corner (Moraceae). Associated vascular plants include *Adiantum gravesii* Hance, *A. malesianum* Ghatak, and *Pteris deltodon* Baker (Pteridaceae).

#### Conservation status.

*Primulina acutiloba* is currently known from a single population at the type locality, comprising 60–80 mature individuals. The species grows on rock faces along a mountain trail. A nearby village at the foot of the mountain results in frequent human activity, as local residents use this trail for grazing cattle and goats and for collecting herbal medicinal plants. Such activities pose a potential threat, as the species may be mistaken for a medicinal herb and removed. Habitat disturbance and individual removal may lead to fluctuations in population size. Following the IUCN Red List Categories and Criteria (IUCN 2025), the species is provisionally assessed as Endangered (EN C2 b).

## Discussion

**Figure 4.**
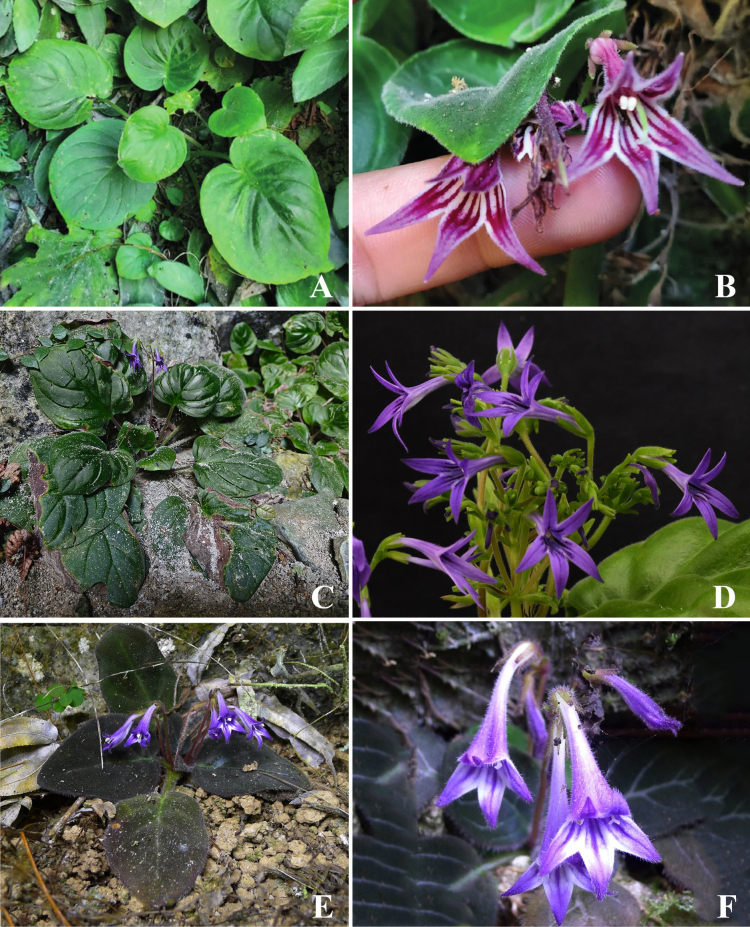
*Petrocodon asterostriatus* (**A, B**), *Pet. integrifolius* (**C, D**), and *Pet. ionophyllus* (**E, F**). **A, C, E**. Plant in natural habitat; **B, D, F**. Flowers. (**A, B, E, F**. Photographed by Fang Wen; **C**. By Chi Xiong; **D**. By De-Chang Meng).

**Figure 5.**
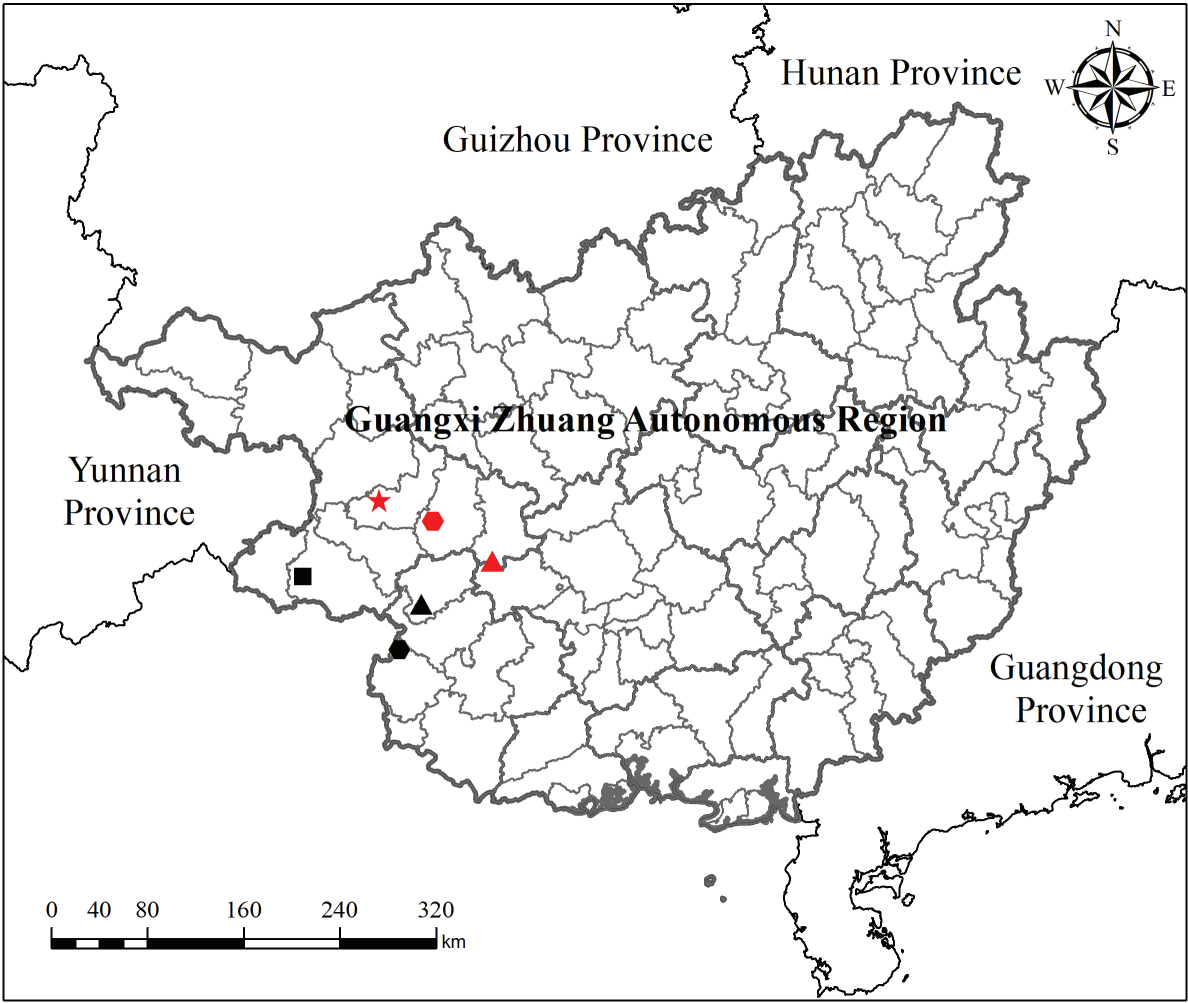
Distribution map of *Primulina acutiloba* (red star), *Pri. pingguoensis* (red triangle), *Pri. pseudoeburnea* (red hexagon), *Petrocodon asterostriatus* (black triangle), *Pet. integrifolius* (black hexagon), and *Pet. ionophyllus* (black square) in China.

The triangular corolla lobes with prominent stripes in *Primulina acutiloba* represent a distinctive morphological feature within the genus, in which oblong, rounded, or obovate lobes are predominant. Such a trait occurs more frequently in the related genus *Petrocodon*. In particular, several *Petrocodon* species distributed in the Baise city and adjacent areas exhibit similar floral morphology, including *Pet. asterostriatus*, *Pet. ionophyllus*, and *Pet. integrifolius* (Figs 4, 5). Notably, both genera co-occur in the Baise region, including the type locality of *Pri. acutiloba*. This sympatric distribution suggests a possible case of convergent evolution of triangular lobes in these lineages, potentially driven by a shared but as yet unidentified pollination syndrome within this specific karst habitat. Clear and consistent morphological differences from its closest relatives, *Pri. pingguoensis* and *Pri. pseudoeburnea* (Table 1), together with its geographically isolated (allopatric) distribution relative to these congeners (Fig. 5), strongly support its recognition as a distinct species.

**Table 1.** Comparison of morphological characters of *Primulina acutiloba*, *Pri. pingguoensis*, and *Pri. pseudoeburnea*.

Characters	*Primulina acutiloba*	*Pri. pingguoensis*	*Pri. pseudoeburnea*
Leaf	narrowly elliptic, elliptic to ovate, 2.2–6.5 × 1.2–3.2 cm, base cuneate to broadly cuneate, apex acute to obtuse	elliptic to broadly ovate, 6.5–9.5 × 4.5–6.5 cm, base slightly oblique, apex obtuse to round	narrowly ovate to elliptic, 2.5–11 × 1.2–4.4 cm, base cuneate, apex acute
Peduncle	3–8 cm long	8–16.5 cm long	10–19 cm long
Bracts	elliptic, 3.5–4.2 mm long	linear or linear-lanceolate, ca. 8 mm long	lanceolate, 15–18 mm long
Calyx lobes	lanceolate, 4.0–4.5 mm long	linear-lanceolate, 4–5 mm long	lanceolate, ca. 11 mm long
Corolla	pale purple, throat with two yellow patches and ca. 15 purple stripes inside, 25–28 mm long; tube nearly cylindric to infundibular, 12–15 × 7–8 mm; lobes triangular, apex acute	pinkish purple, with 8–10 longitudinal dark purple stripes, 16–20 mm long; tube tubular, 10–11 × 6 mm, lobes oblong, apex rounded	purple, with 4 longitudinal lines, ca. 30 mm long; tube narrowly funnelform, ca. 20 × 8 mm, lobes oblong, apex rounded
Disc	ca. 1.2 mm high	ca. 0.8 mm high	ca. 0.5 mm high
Pistil	Pistil 21–24 mm long, ovary 7–8 mm long, style ca. 15 mm long	Pistil 11–15 mm long, ovary 6–8 mm long, style 6–8 mm long	Pistil ca. 20 mm long, ovary ca. 10 mm long, style ca. 10 mm long

The discovery of *Primulina acutiloba* highlights the conservation challenges faced by micro-endemic species in karst ecosystems. The species is currently known from a single population of only 60–80 mature plants within a highly restricted area and is directly threatened by human activities, including frequent trail use and potential harvesting for medicinal purposes. As newly described species inherently possess a higher extinction risk than known species (Liu et al. 2022), *Pri. acutiloba* requires urgent conservation attention. Although ex situ cultivation has been successfully established as a precautionary measure, in situ conservation actions—such as habitat protection and long-term monitoring—are urgently required to prevent potential extinction of this narrowly distributed species.

## Supplementary Material

XML Treatment for Primulina acutiloba

## Additional information

### Use of AI

No use of AI was reported.

### Author ORCIDs

Chi Xiong https://orcid.org/0000-0002-9082-2539

Jia-Xin Fu https://orcid.org/0000-0003-0792-5383

Qi Qin https://orcid.org/0009-0003-1462-8245

Bo Pan https://orcid.org/0000-0003-0996-524X

Hong Liu https://orcid.org/0000-0001-7227-2476

Fang Wen https://orcid.org/0000-0002-3889-8835

### Data availability

All of the data that support the findings of this study are available in the main text.
